# Apical Hypertrophic Cardiomyopathy: A Rare and Hidden Cause of Stroke

**DOI:** 10.7759/cureus.96681

**Published:** 2025-11-12

**Authors:** Immy Stringer, Akshaye Patel, Leyan Edhem, Rebecca Delamere, Gedoni Eni, Adnan Ahmed, Jhiamluka Solano

**Affiliations:** 1 General Internal Medicine, Scunthorpe General Hospital, Scunthorpe, GBR; 2 Medicine, Hull University Teaching Hospitals, Hull, GBR; 3 Internal Medicine, Northern Lincolnshire and Goole NHS Trust, Scunthorpe, GBR; 4 Cardiology, Scunthorpe General Hospital, Scunthorpe, GBR; 5 Internal Medicine, Scunthorpe General Hospital, Scunthorpe, GBR; 6 Cardiology, Hull University Hospital, Castle Hill Hospital, Hull, GBR; 7 Resident Doctor Committee, Royal College of Physicians, London, GBR; 8 Education Committee, Academy of Medical Educators, Cardiff, GBR; 9 Cardiology, York Teaching Hospital NHS Foundation Trust, York, GBR

**Keywords:** acute ischaemic stroke, cardiac magnetic resonance (cmr), electrocardiogram (ecg/ekg), hypertrophic cardiomayopathy, transthoracic echocardiogram

## Abstract

Hypertrophic cardiomyopathy (HCM) is an important but under-recognised cause of cardioembolic stroke, due to impaired myocardial contraction, leading to a region of blood stasis and thus associated thrombus formation. Stroke remains the second leading cause of death worldwide, with increasing incidence even among patients without conventional vascular risk factors and those younger in age. Apical hypertrophic cardiomyopathy (ApHCM), characterised by hypertrophy of the left ventricular apex, is associated with a higher risk of stroke than other HCM variants due to apical cavity obliteration, impaired diastolic filling, and the potential formation of apical aneurysms that serve as a nidus for thrombus. We present the case of a 64-year-old man who attended the emergency department following unilateral peripheral vision loss. He was found to be hypertensive, and a brain MRI confirmed an acute occipital infarct. Subsequent cardiac investigations, including ECG, Holter monitoring, and cardiac MRI, revealed T-wave inversion and evidence of apical hypertrophic cardiomyopathy with an associated subtle aneurysm and an apical thrombus. This case highlights the need to consider ApHCM as a potential cause of ischaemic stroke, where it is typically a less common differential diagnosis. Earlier recognition and individualised management regimens, including counselling and timely anticoagulation, are essential in effectively alleviating symptoms and preventing disease progression.

## Introduction

Stroke is the second leading cause of death globally, with incidence rising due to increased prevalence of metabolic disease, hypertension, and heart disease. In young adults, ischaemic stroke incidence is also rising, with 10-14% of stroke burden attributed to adults <50 years of age, particularly ischaemic strokes [1,2]. Whilst atherosclerotic disease is the leading cause of ischaemic stroke, in the younger population, causes such as arterial dissection, vasculitides, patent foramen ovale, and cardiac thrombus must also be included in the differential for cerebral vascular compromise, often coming into consideration further down the diagnostic pathway.

Hypertrophic cardiomyopathy (HCM) describes a state of enlargement of the left ventricular muscle wall in the absence of hypertension, existing valvular disease, or any systemic condition that could otherwise explain the degree of hypertrophy observed [3]. In adults, a left ventricular wall thickness of >15 mm is used for diagnosis (13 mm if a familial history is present), and it is inherited in an autosomal dominant fashion, with 60% of patients possessing a known pathogenic variant of a sarcomeric gene underlying the muscle’s morphological changes. Of those diagnosed with the HCM phenotype, many have no obvious genetic cause, and even in the presence of a known variant, disease development can still be variable among families. Clinically, it can present with dyspnoea, syncope, or chest pain, whilst a third of patients will not develop outflow obstruction during the disease course, and a high burden of unrecognised disease continues to exist [3].

Apical HCM in particular is characterised by hypertrophy at the left ventricular apex, with apical cavity obliteration in end-systole, apical scarring, aneurysm, and apical perfusion defects [4-6]. Characteristic ECG changes, including large precordial R waves and associated deep T-wave inversion, in conjunction with echocardiography, cardiac MRI, angiography, and stress testing, can aid in diagnosing and assessing severity [3]. The risk profile is different when compared to other variants of HCM, with a notable increase in risk of stroke despite similar overall mortality [4], and evidence of a greater number of adverse events when hypertrophy extends to the ventricular septum [5]. Here, we present the case of a young patient with no significant medical history, diagnosed with apical HCM following an acute ischaemic stroke as his first presentation of the disease.

## Case presentation

A 64-year-old Caucasian male presented to the emergency department following loss of peripheral vision in his right eye. Following specialist assessment, the symptoms had resolved, and the patient denied experiencing any jaw claudication, headache, weakness, sensory changes, or speech disturbance. The eye was unremarkable on examination, with no external abnormality noted. Pupils were equal and reactive, visual fields were preserved, and the anterior segment was unremarkable. The patient had a past ocular history significant for myopia and was otherwise known to have pre-diabetes, hyperlipidaemia, and metabolic dysfunction-associated fatty liver disease. He was taking no regular medications at the time.

**Figure 1:**
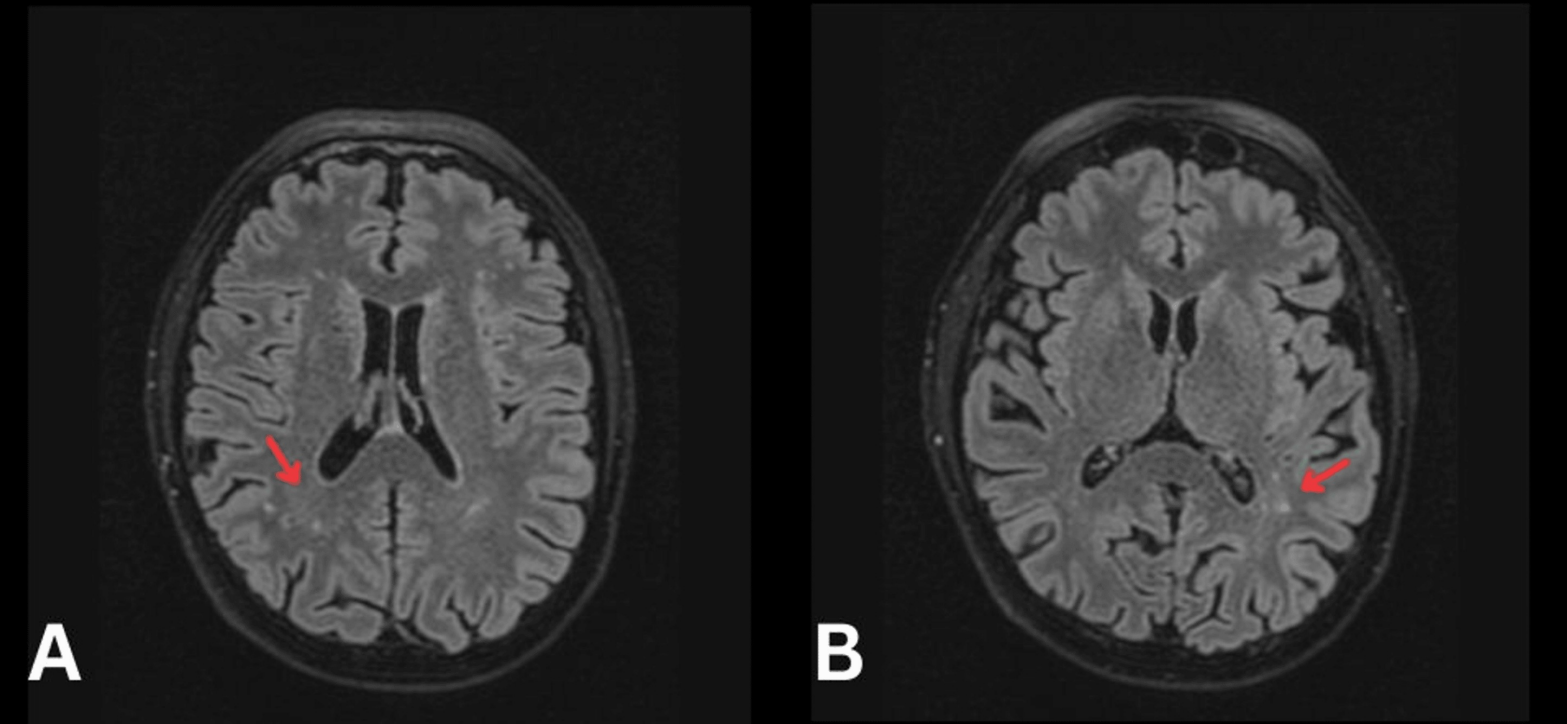
MRI brain T2-weighted imaging A. Right occipital increased signal intensity representing infarct (arrow); B. Left occipital increased signal intensity representing infarct (arrow)

**Figure 2:**
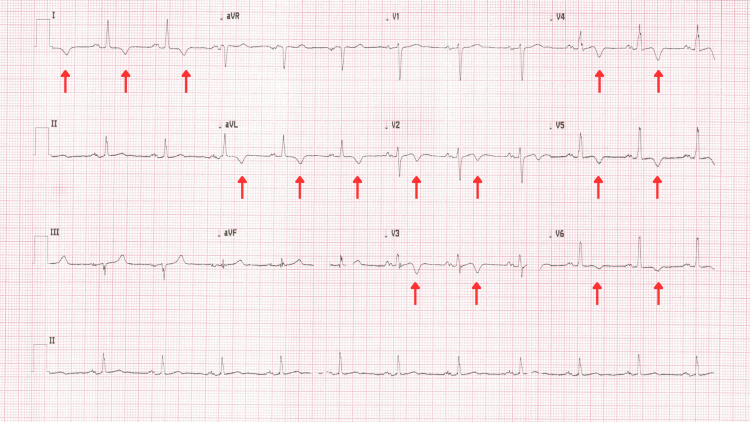
12-lead ECG with evidence of widespread T wave inversion (arrows) ECG: electrocardiogram

Initially, the cause of the symptoms was thought to be related to vascular compromise, and a referral was made to the stroke team. The patient was found to be hypertensive, and blood results showed elevated low-density lipoprotein (LDL) (2.3 mmol/L) and non- high-density lipoprotein (HDL) (3.6 mmol/L), with an glycated hemoglobin (HbA1c) suggestive of pre-diabetes (44 mmol/mol), a normal International Normalized Ratio (INR) (1.0), a mildly elevated troponin (28 ng/L), and platelets (440 10^9/L). MRI of the brain (Figure 1) showed a small focus of acute restricted diffusion involving the left occipital lobe (evidence of acute infarct); echocardiography (Video 1) was reported as unremarkable (with left ventricular ejection fraction visualised as 60‐65%), as was carotid Doppler ultrasound. Electrocardiogram (ECG) (Figure 2) showed T-wave inversion in anterior and lateral leads. 

**Video 1:** (TTE) Transthoracic echocardiogram Left ventricular ejection fraction visualised as 60‐65%.

**Figure 3:**
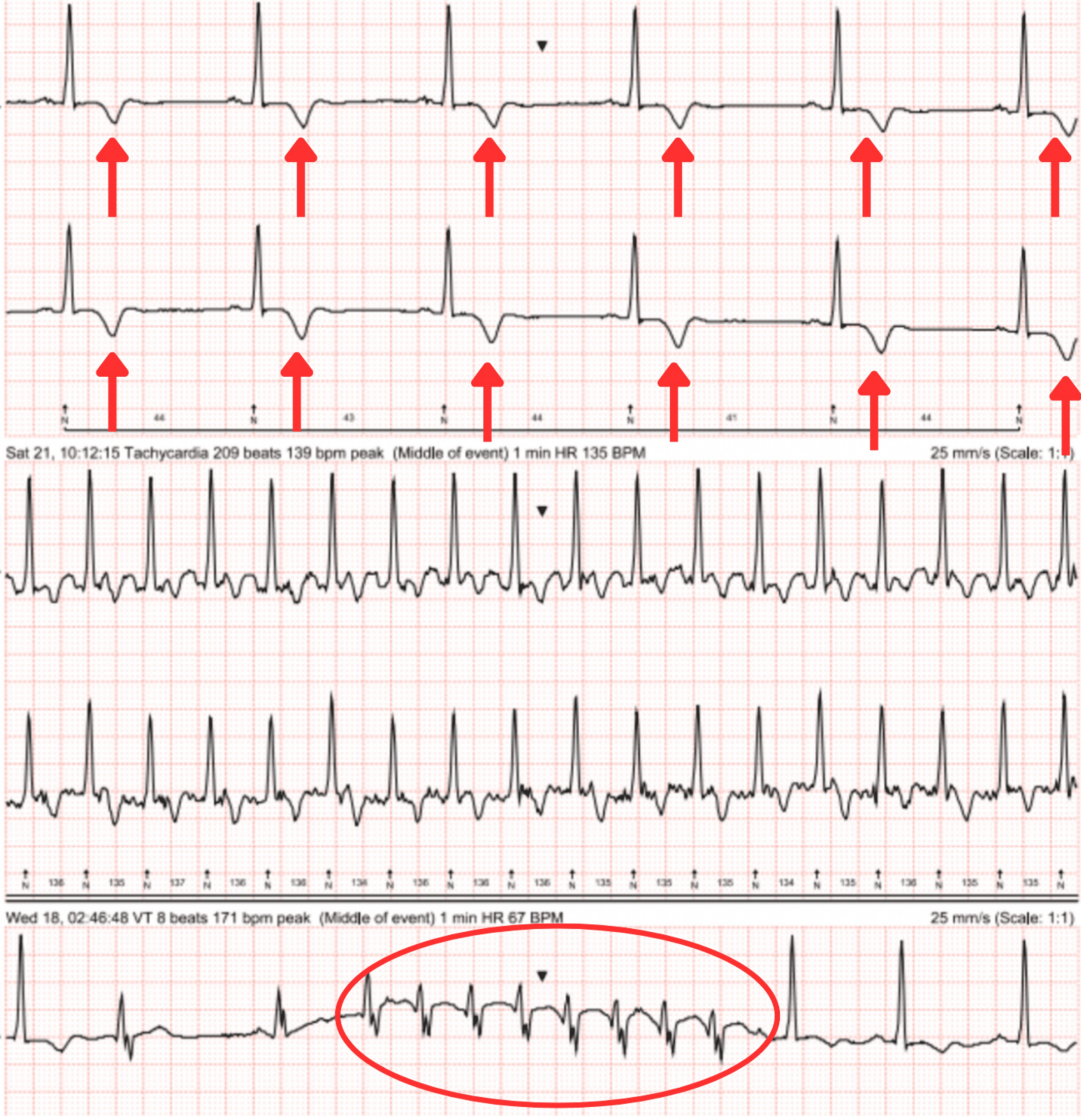
Ambulatory Holter monitoring Top: Recording from the period of bradycardia during monitoring; Middle: Recording from the period of sinus tachycardia; Bottom: Recording capturing eight beats of ventricular tachycardia (red circle), patient asymptomatic. All traces show evidence of consistent deep T wave inversion (arrows).

**Figure 4:**
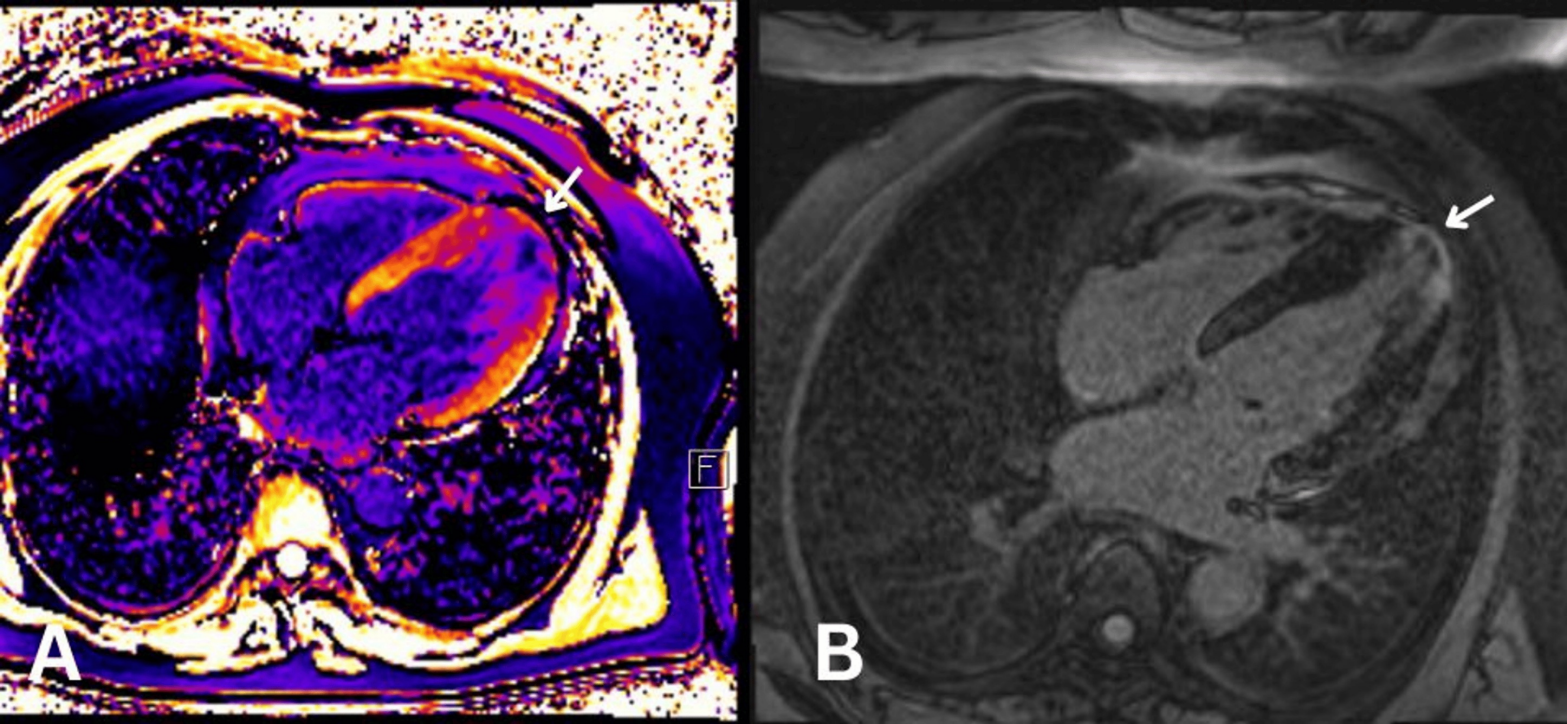
Cardiac MRI There is apical hypertrophy with noted A. T1 mapping showing an apical thrombus (the arrow points at the dark spot) and B. Delayed hyperenhancement showing a transmural post-ischaemic scar (the arrow points at the small ring of gadolinium accumulated in the expanded extracellular space). MRI: Magnetic resonance imaging

Clopidogrel and aspirin therapy were started (with lansoprazole cover) as per guidelines for transient ischaemic attack, alongside atorvastatin, given the raised cardiovascular risk and LDL levels. On follow-up by the ophthalmology team two weeks later, there were no concerns regarding the patient’s vision. Seven-day Holter monitoring and exercise testing were arranged under cardiology; these demonstrated a sinus rhythm with persistent T-wave changes, a <1% ectopic burden, and one episode of broad complex tachycardia lasting eight beats, with the patient asymptomatic during the recording (Figure 3). The patient also achieved a result of 11 minutes with exercise stress testing under the Bruce protocol. Further MRI imaging was arranged, given the ECG abnormalities despite the absence of symptoms. Cardiac MRI revealed apical hypertrophy and evidence of apical hypertrophic cardiomyopathy (Video 2). In addition to this, it showed a transmural postischemic scar with focal akinesia, wall thinning at the level of the apical cap, and an associated subtle aneurysm with an apical thrombus, measuring 15 mm in length and 5 mm in thickness (Figure 4B). 

**Video 2:** Cardiac MRI Normal left and right ventricular size and function with apical hypertrophy. MRI: Magnetic resonance imaging

Anticoagulation in the form of edoxaban 60 mg, based on the patient's choice of anticoagulant instead of warfarin and bisoprolol 2.5 mg, was started, with the atorvastatin dose increased to 40 mg, and clopidogrel 75 mg continued by the stroke team for a short duration of three months. The patient was referred to the inherited cardiac conditions clinic at a tertiary cardiac centre for ongoing investigation and management. The patient remained asymptomatic despite runs of non-sustained ventricular tachycardia, with no family history of sudden cardiac death (SCD), pacemaker or ICD implantation, or known cardiovascular disease. The patient’s European Society of Cardiology (ESC) SCD risk score was 2.4%, and therefore, no implantable cardioverter-defibrillator (ICD) was indicated at this time. The patient was fully cooperative with the follow-up process and understanding of the need to continue with regular surveillance, continue physical activity, reduce alcohol consumption, and be compliant with prescribed medications.

The patient’s family was counselled and offered genetic screening under Leeds (regional genetics centre) in view of apical cardiomyopathy. An ECG on the day of review demonstrated a leftward axis and deep asymmetric T-wave inversion in V3-6, I, and aVL, with an upright T-wave morphology in aVR. The patient is currently under yearly surveillance, given his good clinical condition, with repeat ECG, echocardiogram, 24-hour Holter monitoring, and exercise testing planned for November 2025. Repeat bloods in May 2025 demonstrate improvement in cholesterol (3.5 mmol/L), non-HDL (2.2 mmol/L), LDL (1.7 mmol/L), and HDL (1.3 mmol/L). The patient reported some postural dizziness, believed to have started following the initiation of his new cardiac medications, but continues to deny angina, syncope, palpitations, or dyspnoea out of keeping with that expected of physical activity.

## Discussion

Hypertrophic cardiomyopathy (HCM) describes a state of enlargement of the left ventricular muscle wall that cannot be entirely attributed to loading conditions, such as valvular disease, congenital heart disease, hypertension, or other systemic conditions. Despite being a common genetic heart disease globally, there are a number of diagnostic challenges surrounding HCM and, therefore, apical HCM. Many patients will have no symptoms at the time of diagnosis and are identified incidentally or as a result of screening [1]. This is evident in our case study presented above, whereby our patient’s initial presentation was due to transient loss of vision, and the patient remained asymptomatic despite frequent episodes of tachycardia. 

Apical hypertrophic cardiomyopathy (ApHCM) specifically presents diagnostic challenges compared to more common and better-understood HCM variants. The definition of ApHCM, as the name suggests, is wall thickness in the apex >15mm and a ratio of maximal apical to posterior wall thickness ≥1.5mm (or >1.3mm in certain criteria) based on echocardiography or cardiovascular magnetic resonance imaging (CMR) [7,8]. It is predominantly characterised by localised hypertrophy confined to the left ventricular (LV) apex. First described in Japan, where it constitutes up to a quarter of HCM cases, its prevalence in Western populations remains significantly lower, estimated at 1-3% of HCM cohorts [9,10].

The pathophysiology of ApHCM involves asymmetric hypertrophy, leading to increased wall stress and impaired diastolic function. Unlike obstructive forms of HCM, ApHCM generally spares the outflow tract but may still result in elevated LV end-diastolic pressures and impaired coronary microvascular function, predisposing to subendocardial ischaemia [11]. In echocardiographic studies of left ventricular wall thickness, it is essential that all LV segments, from base to apex, are examined; this can prove challenging, particularly at the apex. Clinicians must carefully assess this area using parasternal long- and short-axis views with apical views to detect ApHCM. In patients with ECG changes but non-diagnostic echocardiography, additional imaging should be performed. CMR may better detect ApHCM in comparison to echocardiography, as 40% of cases were missed by echocardiography, which were later detected with CMR [12]. In addition, CMR is more sensitive in detecting apical aneurysms [13], again evident in our case study, where echocardiography was unremarkable, but CMR revealed apical hypertrophy and thrombus. Cardiac magnetic resonance imaging (CMR) has proven invaluable in delineating apical morphology and identifying myocardial fibrosis through late gadolinium enhancement, which may have prognostic implications [14].

Despite often being considered a more "benign" subtype, the risk profile of ApHCM is increasingly recognised as heterogeneous. While many patients remain asymptomatic or experience mild symptoms, complications such as atrial fibrillation, apical aneurysm formation, and ventricular arrhythmias have been reported. Notably, the presence of an apical aneurysm is associated with increased thromboembolic risk and sudden cardiac death (SCD), necessitating careful longitudinal monitoring [15]. Larger aneurysms (>2 cm) and those with thin or dyskinetic walls carry greater risks of thrombus formation and ventricular arrhythmias. Late gadolinium enhancement on CMR correlates with arrhythmic risk, suggesting that myocardial fibrosis may serve as a substrate for malignant ventricular tachyarrhythmias [15]. Therefore, risk stratification in ApHCM requires a comprehensive approach that includes imaging, symptom burden, and rhythm surveillance, particularly in individuals with apical aneurysms or extensive fibrosis.

Applying Cine steady-state free precession (SSFP) MRI imaging is superior for thorough morphological assessment of cardiac function, and using a ‘no limited’ view can define all phenotypes of HCM [16]. For our case, notable CMR findings include apical hypertrophy, transmural postischemic scar, apical cap wall thinning, and an associated subtle aneurysm with apical thrombus. Cardiac computed tomography (CT) may be used as an alternative when TTE is technically limited and CMR is contraindicated. Cardiac CT can clearly visualise left ventricular structure and function with a high specificity and sensitivity in detecting coronary artery stenosis in ApHCM patients with evidence of myocardial ischaemia [5]. Through coronary angiography, we can exclude coronary disease and evaluate the left ventricular cavity, with characteristic features including an end-diastolic ‘ace of spade’ sign and congenital coronary artery anomalies [8].

Management of ApHCM aims to improve functional capacity, alleviate symptoms, and prevent disease progression/complications. Medical management involves initiating beta-blocker or calcium channel blocker drug therapy in patients with preserved ejection fraction, and these should be titrated to their effectiveness. For patients with reduced ejection fraction, standard heart failure medication should be initiated with caution to prevent hypovolaemia and hypotension. The use of implantable cardiac defibrillators (ICD) should be considered for patients with high-risk factors for sudden cardiac death, despite this risk being lower in ApHCM compared to those with the typical HCM phenotype [17]. For patients who are refractory to maximal medical treatment, highly specialist surgical options exist, such as transapical myectomy, which significantly increases end-diastolic volume and stroke volume and enlarges the LV cavity [18].

Stroke in apical hypertrophic cardiomyopathy (ApHCM) can arise through multiple mechanisms. ApHCM causes apical wall thickening and, in our case, post-ischaemic scarring and akinesia, which all compromise the normal contraction of the apical myocardium. These abnormalities create a region of stasis in blood flow, especially during end-systole, which can result in apical cavity obliteration [7]. In the presence of myocardial fibrosis, this serves as a risk for thrombus formation and, thus, an elevated stroke risk [19]. 

Atrial fibrillation (AF) is a well-established risk factor for stroke in all subtypes of HCM, including ApHCM [20]. However, in the presented case, the stroke was most likely cardioembolic secondary to an apical mural thrombus. Unlike a thrombus originating from the atrium, mural thrombi in the left ventricle originate from regions of akinetic or aneurysmal myocardium, particularly in the apex. Apical perfusion defects are nearly universal in ApHCM and are strongly associated with progressive fibrosis, which increases the risk of thromboembolism [4]. In our case, the apical thrombus likely embolised via the left ventricular outflow tract and aorta to the posterior cerebral circulation. The MRI demonstrated a focal infarct in the left occipital lobe, consistent with an artery embolism occluding a distal branch of the posterior cerebral artery. The patient’s transient loss of peripheral vision and normal carotid Doppler findings further support this, in contrast to atherosclerotic or carotid thromboembolic aetiology.

Management of stroke and anticoagulation in ApHCM remains complex, particularly in patients without atrial fibrillation (AF) but with anatomical risk factors for thromboembolism. Current European Society of Cardiology (ESC) and American Heart Association (AHA) guidelines recommend anticoagulation in HCM patients with AF or atrial flutter and in those with documented left ventricular thrombus or an apical aneurysm [3,21]. However, there is no specific guidance regarding anticoagulation in ApHCM patients in sinus rhythm without an aneurysm but with other high-risk features such as apical scarring or akinesia. This emphasises the need for individualised risk stratification and further research to inform evidence-based management in this group of patients. In ApHCM with documented thrombus or aneurysm, long-term anticoagulation is generally recommended given the persistent substrate for stasis and embolisation. If a thrombus resolves and an aneurysm remains stable, indefinite anticoagulation may still be reasonable based on individual risk factors such as fibrosis, akinesia, or aneurysm morphology.

Genetic testing is increasingly recommended when apical hypertrophic cardiomyopathy (ApHCM) is suspected; molecular confirmation sharpens diagnosis and enables cascade screening [21]. Commonly identified sarcomeric mutations in ApHCM are similar to those from classical HCM and include MYBPC3 and MYH7, the primary genes involved [22,23]. These are inherited in an autosomal dominant pattern, as observed in classical HCM. Other gene mutations identified from the literature include ACTC1, MYL2, MYL3, TTNT2, TTNI3, TNNC1, and TPM1 [22,24].

Positive genotyping, however, is less common with ApHCM. Several studies have demonstrated a detection rate of pathogenic genetic variants between 14% and 25% [24,22] as opposed to around 40% for classical HCM [21]. Genome-wide studies show that many cases involve polygenic inheritance, where multiple small-effect variants collectively increase disease risk rather than a single dominant mutation [21]. Furthermore, cardiomyopathies likely exist on a genetic spectrum, from cases caused by rare, high-impact variants to more complex, highly polygenic forms, with overlapping modifiers influencing disease severity and expression [21,24]. A recent study [23] found that detection of pathogenic variants in sarcomeric protein-encoding genes among their ApHCM cohort was greater in the distal-dominant than pure apical forms, suggesting that complex genetics alongside gene-environment interactions may be responsible for this phenotype in specific individuals.

Likewise, studies on clinical outcomes between genotype-positive and genotype-negative cases of ApHCM have offered conflicting results. Towe and colleagues found no statistically significant difference in adverse outcomes between genotype-positive and genotype-negative ApHCM patients [22], in contrast to studies around typical HCM [25,26]. Disparities between studies may have been due to the inclusion of non-cardiac-related death in the former; nonetheless, a recent study among ApHCM patients [23] revealed a trend towards worse non-fatal cardiac adverse outcomes in the variant-positive group as well as in the distal-dominant form of ApHCM. This suggests that genotype positivity may, in fact, predict a worse prognosis for this cohort and could inform risk stratification and ICD implantation counselling.

As per the ESC guidelines [21], all first-degree relatives of cardiomyopathy patients should have ECG and cardiac imaging screening. If a causative genetic variant is found, relatives should pursue genetic testing, and those without the variant can usually be discharged with advice to return if symptoms develop. Relatives who carry the variant need regular follow-up with ECG, imaging, and other tests as needed. If no genetic variant is identified, ongoing clinical monitoring is recommended for all first-degree relatives, especially children, due to age-related disease onset. In families with only one affected person and no identified variant, follow-up can be less frequent. Pre- and post-test genetic counselling is also recommended to gather further family history and determine the probability of genetic disease, as well as help patients and families understand the psychological, social, professional, ethical, and legal implications of a genetic diagnosis.

Regarding long-term follow-up, the ESC guidelines [21] suggest that patients with cardiomyopathy need lifelong follow-up to monitor symptoms, arrhythmias, and heart function. Clinical exams with ECG and echocardiography should be done every 1-2 years, or sooner if new symptoms arise, and an ambulatory ECG is recommended every 1-2 years to detect silent arrhythmias. Cardiac MRI is advised every 2-5 years, while exercise testing can be done every 2-3 years unless symptoms worsen. 

## Conclusions

Given the increasing incidence of ischaemic stroke in young individuals, it is essential to consider less common differential diagnoses, particularly ApHCM, earlier in the diagnostic process. When first-line investigations yield inconclusive results and the diagnosis remains uncertain, more specific imaging techniques, such as cardiac MRI, should be employed for both diagnosis and prognosis. Additionally, genetic testing should be integrated into the diagnostic pathway as soon as ApHCM is suspected. Regardless of the presence of a causative genetic variant, genetic counselling and ongoing clinical monitoring are crucial for first-degree relatives. Through the use of individualised risk stratification, we can personalise treatments and management strategies on a patient-to-patient basis, effectively alleviating symptoms and preventing disease progression.
